# T-RMSD: a web server for automated fine-grained protein structural classification

**DOI:** 10.1093/nar/gkt383

**Published:** 2013-05-28

**Authors:** Cedrik Magis, Paolo Di Tommaso, Cedric Notredame

**Affiliations:** ^1^Bioinformatics and Genomics Programme, Centre For Genomic Regulation (CRG), Carrer del Doctor Aiguader 88, 08003 Barcelona, Spain and ^2^Universitat Pompeu Fabra (UPF), 08003 Barcelona, Spain

## Abstract

This article introduces the T-RMSD web server (tree-based on root-mean-square deviation), a service allowing the online computation of structure-based protein classification. It has been developed to address the relation between structural and functional similarity in proteins, and it allows a fine-grained structural clustering of a given protein family or group of structurally related proteins using distance RMSD (dRMSD) variations. These distances are computed between all pairs of equivalent residues, as defined by the ungapped columns within a given multiple sequence alignment. Using these generated distance matrices (one per equivalent position), T-RMSD produces a structural tree with support values for each cluster node, reminiscent of bootstrap values. These values, associated with the tree topology, allow a quantitative estimate of structural distances between proteins or group of proteins defined by the tree topology. The clusters thus defined have been shown to be structurally and functionally informative. The T-RMSD web server is a free website open to all users and available at http://tcoffee.crg.cat/apps/tcoffee/do:trmsd.

## INTRODUCTION

Modern biology has long relied on comparisons to identify and quantify patterns revealing the nature of evolutionary forces. The recent availability of large amounts of molecular data has not changed this paradigm (1). Phylogenetics are based on the notion that evolutionary history can be reconstructed using sequence comparisons to estimate the number of mutations accumulated through long periods. If one assumes the existence of some evolutionary clock, these divergence rates can be turned into evolutionary distances and can be used to reconstruct phylogenetic trees. In practice, however, evolutionary reconstruction is hampered by many confounding factors, the main one being a phenomenon known as multiple sampling. Multiple sampling results from the possibility that a given site may have mutated more than once and possibly reverted to its original state, thus resulting in inaccurate distance estimations. Statistical models can be used to partly correct for this effect, but the reliability of these corrections decreases when evolutionary distances increase. The most obvious way to address this issue is to take advantage of sequence-encoded signals (i.e. protein-coding potential) that diverge slower than nucleotide sequences because of higher-order functional and structural constraints. Yet, even though they increase the ‘look-back time’, protein sequences do not entirely solve the multiple sampling problem and merely shift it to larger evolutionary distances. Nevertheless, new issues arise when dealing with protein sequences <30% identity, entering the so-called ‘twilight zone’ (2); in this range of identity, establishing accurate protein alignment models becomes challenging.

Our inability to align remote homologs makes it virtually impossible to detect, from sequence alone, regularities that may reveal functional similarities across distantly related proteins or structural features supporting such functions. This well-known issue has long been addressed through the systematic usage of structural information, or more recently through the usage of homology extension methods, such as PROMALS (3) or PSI-Coffee (4). Back in 1980, it has been shown (5) that one can derive functionally meaningful protein classification by considering structural properties alone. It is now well established that structural properties constitute the most resilient character encoded by genomic DNA. Given an accurate alignment that establishes the correspondence between homologous amino acids, protein structures can, therefore, be compared even when their underlying sequences have diverged well passed the saturation point. This observation has triggered the development of several structural classification schemes, such as SCOP (6), CATH (7) or the Dali Domain Dictionary (8). These resources all constitute an attempt to derive highly informative classifications by grouping all available structures through clustering meant to bring together structurally homologous protein sequences, and they can be seen as the most similar online tools to the one we describe here. These classifications, however, as informative as they may be, have two limitations: they are based on pairwise comparisons, and they do not provide any estimate of the robustness of the nodes supporting each individual classification. Furthermore, these classifications may be poorly informative when dealing with closely related structures, as they tend to produce unresolved nodes. The T-RMSD method (tree-based on root-mean-square deviation) (9), deployed in this new web server, is an attempt to derive structure-based clusterings having a resolution similar to their sequence-based phylogenetic counterparts.

So far, T-RMSD has been applied and validated on three functionally well-described protein families: the small GTPase RAS families (closely related homologs, ∼50% identity), the tumor necrosis factor (TNF) superfamily (distantly related homologs, ∼30%) and the cysteine-rich domains associated with the TNF receptor superfamily (distantly related homologs, ∼25%) (9,10). The T-RMSD was either able to recapitulate a sequence-based clustering (for closely related homologs) or able to derive a new clustering (for distantly related homologs) functionally more informative than a mere sequence-based phylogenetic reconstruction. These analyses are encouraging and bringing us one step closer to a set-up that will make it possible to show whether structural data constitute a highly resilient character suitable for phylogenetic reconstruction, especially for families exhibiting low-sequence conservation. If this could be shown, it would make a method like T-RMSD the procedure of choice for the reconstruction of deep nodes in large phylogenies. As for now, the method has only been shown to be suited for the identification of functional subclasses among sets of related sequences with known structures.

## ALGORITHM

Given a set of homologous sequences with known structures, the T-RMSD (9) is a method designed to turn the multiple sequence alignment (MSA) of these sequences into a structure-based clustering. This clustering is estimated through the systematic comparison of intramolecular distances, in a way similar to the DALI algorithm (11).

The procedure implemented in the web server is a pipeline similar to the one described previously (9,10). It first requires the assembly of a high-accuracy MSA. In practice, we use the Expresso mode of T-Coffee (12,13). T-Coffee is a multiple sequence aligner able to generate an MSA by the combination of any third party pairwise or multiple alignment method. Expresso is a special mode of T-Coffee able to generate the MSA using a combination of sequences and structures. Expresso can be described as a template-based multiple sequence aligner, where templates are the experimental structures of the considered sequences. Expresso uses BLAST to fetch from the Protein Data Bank [PDB (14)] the structures corresponding to each of the sequences in the data set. It then uses the T-Coffee multiple alignment algorithm combined with a structure-based pairwise aligner [SAP by default (15)] to estimate the final structural alignment. Sequences for which no structure can be found are simply ignored from subsequent analysis.

This alignment and the associated structures are then fed to the T-RMSD so that the cluster can be computed. This analysis can only be carried out if the MSA of the sequences with known structures contains at least two ungapped columns (i.e. columns with one aligned residue across all considered sequences). The final clustering, which has the aspect of a phylogenetic tree, is achieved through a combination of structure comparison and tree reconstruction methods. First of all, a distance matrix is estimated on each ungapped column by measuring the differences of distance RMSD (dRMSD) (16) between pairs of residues contained in the considered column and all other ungapped columns. Distance matrices thus derived (one for each ungapped column) are then turned into a set of trees (one per column) using a clustering algorithm. We use the PHYLIP neighbor-joining implementation (17,18), but any algorithm able to handle distance matrices could be used. The resulting collection of trees is eventually combined into a consensus tree using the Consense application (majority rule) from the PHYLIP package. The result is a binary unrooted tree, where each node is associated with a value that indicates the number of supporting ungapped positions.

## T-RMSD WEB SERVER

The T-RMSD web server is a part of the T-Coffee web platform (13) and is accessible directly from http://tcoffee.crg.cat/apps/tcoffee/do:trmsd. It does not require any login and is compliant with all major web browsers (Mozilla Firefox 5+, Google Chrome, Internet Explorer 8+, Safari 6+ and Opera 11+). Results can be retrieved from the web server or received by email, provided the users had given their email address; it is especially advisable when submitting computationally expensive jobs. It can deal with data sets of up to 150 sequences. All the functions of the server are also available through the command line version of T-Coffee (freeware open source, GPL license) that can be downloaded from www.tcoffee.org.

### Using the T-RMSD web server

As described in the previous section, the T-RMSD web server is a two-step pipeline that starts by deriving an Expresso structure-based MSA on which it applies the T-RMSD algorithm. The default procedure only requires as input a data set of unaligned homologous protein sequences in a standard format (Fasta or ClustalW). More advanced options are available, essentially related to some extra control of the alignment procedure. In this advanced mode, users can select the aligner of their choice, provide their own structures and template files or modify the format of the resulting alignment. All available features can be accessed and selected by simply displaying the extra options menu. The whole process can be reproduced by installing a local version of T-Coffee and running consecutively the two required T-Coffee methods: Expresso and T-RMSD.

### Use of PDB structures

The selection of proper PDB templates for the provided sequences is a critical step. By default, the server uses as templates PDB files of sequences having at least 80% identity with the considered sequences. There is no filter or parameter regarding the quality of the structure file identified through BLAST, therefore, the structure might not be optimal regarding its coverage to the query sequence (i.e. unresolved regions lacking electronic density) or its resolution. If a template is not satisfying, users have the possibility to upload up to three structures to substitute such unsatisfying structures. The template file must be modified accordingly by replacing the template names and then uploaded to the web server (see in ‘Output Interpretation’ section (8), mode ‘Replay’). Also, the structure format recognized by the web server is standard, thus using modified PDB files or homemade structures should strictly follow the PDB format. An alternative would be to modify or reformat structure files using T-Coffee re-formatting options in its command line mode using a local installation of the T-Coffee package on your own system. Users wishing to use a larger number of private structures are encouraged to install the package locally.

### Output interpretation

**Figure 1.**
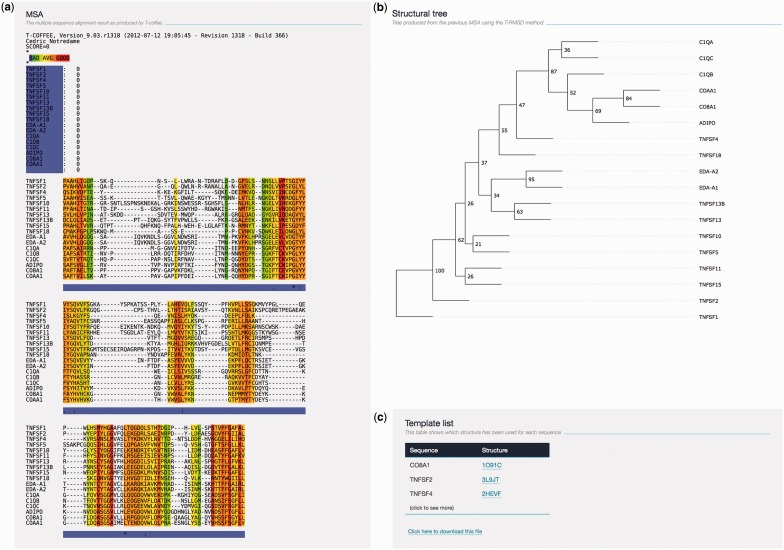
Sample T-RMSD result page. (**a**) The MSA colored according to T-RMSD color scheme where ungapped columns in red correspond to highly discriminant positions and blue correspond to poorly discriminant one. The color scheme represents the individual contribution to each ungapped column in generating the final clustering: positions structurally highly conserved among all proteins will have low contribution in discriminating structural variations. For post-processing purpose, users are advised to download the text-based version of the alignment available in the ‘Result File’ section. (**b**) The structural clustering generated by the T-RMSD method displayed using the jsPhyloSVG library. The values at each node, analog to bootstrap values, represent the support of the split. (**c**) The template section where the template file and the list of structure files used for the computation can be visualized.

The server final output is a summary page containing all results files and parameters used for the run. With large structures, the computation time can be significant, and users are encouraged (but not required) to provide their email; therefore, they can be notified when the computation is finished. The summary displays the following items in order:

1. **‘MSA’**: the MSA colored according to the T-RMSD scheme (Figure 1a). The MSA colored according to the T-Coffee consistency scheme can be downloaded from the section (4) ‘Result Files’. The T-RMSD color scheme is related to the informativeness of each ungapped column, from blue (poorly discriminant) to red (highly discriminant). The term discriminant reflects the contribution of a given ungapped column to the final tree topology.
2. **‘Structural tree’**: the resulting structural tree with its support values (Figure 1b). The tree is rendered on the HTML page using the jsPhyloSVG library (19). Optionally, the tree can be displayed in a separate interactive window using the PhyloWidget application (20). The users can also download the tree file in Newick format to use other visualization tools (see ‘Visualizing Tree’ section).
3. **‘Template list’**: the list of template files associated with each sequence (Figure 1c). The structure files are named after the PDB code and the chain identifier (Chain ID) of the structure used for the computation. Structures are linked to their corresponding PDB webpage for more information.
4. **‘Result files’**: the result files coming from the alignment and T-RMSD method. All files can be downloaded as a single zip file.
5. **‘Citation’**: the related article when citing the T-RMSD.
6. **‘Info’**: the information related to the current job.
7. **‘Command line’**: the command lines (separated by a semicolon) used by the web server to run successively the Expresso alignment and the T-RMSD method. The command lines can then be reused and/or modified to run on a local installation of the T-Coffee package on your own system.
8. **‘Replay’**: this feature allows the users to re-run the job while modifying input options or data.
9. **‘Feedback’**: the support and comment section. The users can contact the developer team for any suggestion, comment or problem.

### Visualizing trees

The development of visualization tools for phylogenetic trees is nearly a field on its own. The T-RMSD web server does not provide such a tool but it uses the jsPhyloSVG JavaScript library to provide a simple visualization of the consensus structural tree generated by the T-RMSD method (Figure 1b). Moreover, an interactive editable version of the same tree is available by integrating the PhyloWidget application. Users can also download the Newick formatted tree and use a tree visualization tool of their choice [iTOL (21), ETE (22) or ATV (23) being among the most widely used]. These tools are not developed by the T-Coffee developer team; thus, choice and usage of these tools are the responsibility of the user.

## CONCLUSION

We describe the T-RMSD web server, a new fine-grained structural clustering method available within the T-Coffee web server. Given a set of structurally related proteins or protein families, T-RMSD will generate a supported structural clustering. The tree is supported by accuracy estimates analog to bootstrap values in phylogenetic reconstruction. We have previously demonstrated the capacity of T-RMSD to associate structural clusters thus generated with biologically relevant features for several families of particular biological interest. Our interesting findings justify a more systematic usage of such an approach to refine or validate protein sequence and functional annotation, and in the future to evaluate the possibility of reconstructing deep phylogenies.

## FUNDING

Plan Nacional [BFU2008-00419] is financed by the Spanish Ministry of Economy and Competitiveness (MINECO) and Quantomics [KBBE-2A-222664] is financed by the 7th Framework Programme of the European Commission (to C.N., P.D. and C.M.); Computational resources are provided by the Center for Genomic Regulation (CRG) of Barcelona and the Vital-IT infrastructure of the Swiss Institute of Bioinformatics. Funding for open access charge: Plan Nacional [BFU2008-00419]; and Quantomics [KBBE-2A-222664].

*Conflict of interest statement*. None declared.

## References

[1] Breen MS, Kemena C, Vlasov PK, Notredame C, Kondrashov FA (2012). Epistasis as the primary factor in molecular evolution. Nature.

[2] Sander C, Schneider R (1991). Database of homology-derived protein structures and the structural meaning of sequence alignment. Proteins.

[3] Pei J, Grishin NV (2007). PROMALS: towards accurate multiple sequence alignments of distantly related proteins. Bioinformatics.

[4] Chang JM, Di Tommaso P, Taly JF, Notredame C (2012). Accurate multiple sequence alignment of transmembrane proteins with PSI-Coffee. BMC Bioinformatics.

[5] Lesk AM, Chothia C (1980). How different amino acid sequences determine similar protein structures: the structure and evolutionary dynamics of the globins. J. Mol. Biol..

[6] Murzin AG, Brenner SE, Hubbard T, Chothia C (1995). SCOP: a structural classification of proteins database for the investigation of sequences and structures. J. Mol. Biol..

[7] Pearl FM, Bennett CF, Bray JE, Harrison AP, Martin N, Shepherd A, Sillitoe I, Thornton J, Orengo CA (2003). The CATH database: an extended protein family resource for structural and functional genomics. Nucleic Acids Res..

[8] Dietmann S, Park J, Notredame C, Heger A, Lappe M, Holm L (2001). A fully automatic evolutionary classification of protein folds: Dali Domain Dictionary version 3. Nucleic Acids Res..

[9] Magis C, Stricher F, van der Sloot AM, Serrano L, Notredame C (2010). T-RMSD: a fine-grained, structure-based classification method and its application to the functional characterization of TNF receptors. J. Mol. Biol..

[10] Magis C, van der Sloot AM, Serrano L, Notredame C (2012). An improved understanding of TNFL/TNFR interactions using structure-based classifications. Trends Biochem. Sci..

[11] Holm L, Sander C (1993). Protein structure comparison by alignment of distance matrices. J. Mol. Biol..

[12] Poirot O, Suhre K, Abergel C, O’Toole E, Notredame C (2004). 3DCoffee@igs: a web server for combining sequences and structures into a multiple sequence alignment. Nucleic Acids Res..

[13] Di Tommaso P, Moretti S, Xenarios I, Orobitg M, Montanyola A, Chang JM, Taly JF, Notredame C (2011). T-Coffee: a web server for the multiple sequence alignment of protein and RNA sequences using structural information and homology extension. Nucleic Acids Res..

[14] Bernstein FC, Koetzle TF, Williams GJ, Meyer EF, Brice MD, Rodgers JR, Kennard O, Shimanouchi T, Tasumi M (1977). The Protein Data Bank. A computer-based archival file for macromolecular structures. Eur. J. Biochem..

[15] Taylor WR, Orengo CA (1989). Protein structure alignment. J. Mol. Biol..

[16] Nishikawa K, Ooi T (1974). Comparison of homologous tertiary structures of proteins. J. Theor. Biol..

[17] Saitou N, Nei M (1987). The neighbor-joining method: a new method for reconstructing phylogenetic trees. Mol. Biol. Evol..

[18] Felsenstein J (2005). PHYLIP (Phylogeny Inference Package) version 3.6.

[19] Smits SA, Ouverney CC (2010). jsPhyloSVG: a javascript library for visualizing interactive and vector-based phylogenetic trees on the web. PLoS One.

[20] Jordan GE, Piel WH (2008). PhyloWidget: web-based visualizations for the tree of life. Bioinformatics.

[21] Letunic I, Bork P (2011). Interactive Tree Of Life v2: online annotation and display of phylogenetic trees made easy. Nucleic Acids Res..

[22] Huerta-Cepas J, Dopazo J, Gabaldon T (2010). ETE: a python environment for tree exploration. BMC Bioinformatics.

[23] Zmasek CM, Eddy SR (2001). ATV: display and manipulation of annotated phylogenetic trees. Bioinformatics.

